# The complete mitochondrial genome of *Triplophysa alticeps*

**DOI:** 10.1080/23802359.2016.1202738

**Published:** 2016-09-05

**Authors:** Le Wang, Pei-Xian Luan, Ping-Ping Wang, Qiu-Shi Wang, Jian-Guo Lu

**Affiliations:** Heilongjiang River Fisheries Research Institute, Chinese Academy of Fishery Sciences, Harbin, China

**Keywords:** Genome, mitochondrial, *Triplophysa alticeps*

## Abstract

The complete mitochondrial genome of *Triplophysa alticeps* was 16,569 bp; it contains 13 protein-coding genes, 22 transfer RNA genes, 2 ribosomal RNA genes, and a D-loop. It is a circular molecule with a typical gene arrangement of vertebrate mitochondrial DNA. This study could be provided insights into the evolution of *Osteichthyes* mitochondrial genomes.

*Triplophysa alticeps* belongs to Nemacheilinae subfamily. *Triplophysa* is one of the largest genera of stone loaches, over 130 species distributed through the whole of central Asia to the southwest of China, such as Qaidam Basin and Qinghai Lake (Prokofiev 2006; Froese & Pauly 2014). In this study, the complete sequence of the mitochondrial genome of *T. alticeps* was isolated (GenBank accession number is KX239473). *Triplophysa alticeps* was collected from Qinghai Lake, northwestern of Qinghai Province, China. The total genomic DNA was extracted with the phenol–chloroform method (Taggart et al. 1992). The disposition of DNA sample was stored in Heilongjiang River Fisheries Research Institute, Chinese Academy of Fishery Sciences, and the accession number is hrfri2016018. Forty primers designed by the Primer 5.0 software were used to amplify the target PCR products for sequencing, and the PCR products were analyzed by ContigExpess software (CA). The location of protein-coding genes and rRNA genes were compared with the known sequence of other teleost species by BLAST (http://blast.ncbi.nlm.nih.gov/Blast.cgi). Furthermore, the transfer RNA (tRNA) sequences were identified using the program tRNAscan-SE 1.21 (http://lowelab.ucsc.edu/tRNAscan-SE/).

The complete mitochondrial DNA of *T. alticeps* was 16,569 bp. The base count of the constitution was 27.11% for A, 25.69% for C, 19.11% for G, 28.09% for T. Overall, the whole mitochondrial comprise of 13 protein-coding genes, 22 transfer RNA genes (22tRNA), 2 ribosomal RNA genes (two rRNA), and a D-loop. The neighbour-joining phylogenetic tree was constructed based on the sequence of CYTB of *T. alticeps* and other teleosts, the result showed that *T. anterodorsalis* and *T. bleekeri* had higher homology compared to *T. alticeps* (Figure 1).

**Figure 1.**
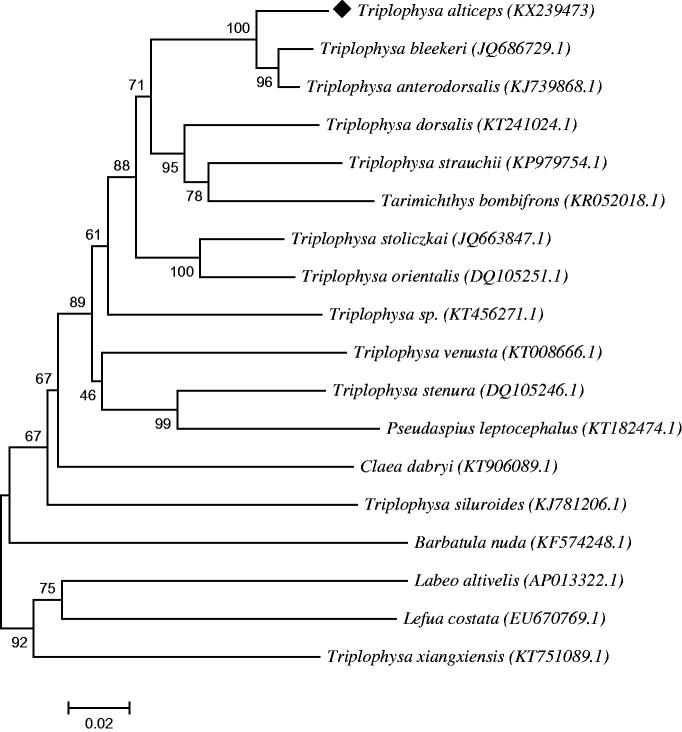
Phylogenetic tree constructed of CYTB between *T. alticeps* and other teleosts. It was constructed by the neighbour-joining method.
